# Mortality Risk Stratification Using Cluster Analysis in Patients With Myositis-Associated Interstitial Lung Disease Receiving Initial Triple-Combination Therapy

**DOI:** 10.3389/fmed.2022.883699

**Published:** 2022-05-09

**Authors:** Takahisa Gono, Kenichi Masui, Shinji Sato, Masataka Kuwana

**Affiliations:** ^1^Department of Allergy and Rheumatology, Nippon Medical School Graduate School of Medicine, Tokyo, Japan; ^2^Scleroderma/Myositis Center of Excellence, Nippon Medical School Hospital, Tokyo, Japan; ^3^Department of Anaesthesiology, Yokohama City University Hospital, Yokohama, Japan; ^4^Division of Rheumatology, Department of Internal Medicine, Tokai University School of Medicine, Isehara, Japan

**Keywords:** interstitial lung disease, polymyositis (PM), dermatomyositis (DM), anti-MDA5 antibody, cluster analysis, triple-combo therapy

## Abstract

**Objective:**

To stratify patients with polymyositis/dermatomyositis-associated interstitial lung disease (ILD) who were initially treated with an intensive regimen consisting of high-dose corticosteroids, a calcineurin inhibitor, and intravenous cyclophosphamide (triple-combo therapy) into subgroups based on mortality outcomes by a cluster analysis using a large-scale multicenter retrospective cohort of Japanese patients with myositis-associated ILD (JAMI).

**Methods:**

Two-step cluster analysis of preclustering and subsequent hierarchical clustering was conducted in 185 patients who received triple-combo therapy in an unbiased manner. Initial predictors for mortality previously reported in patients with myositis-associated ILD were used as variables and included age, sex, disease duration, classification of myositis, requirement of supplemental oxygen, anti-aminoacyl tRNA synthetase (ARS) antibody, anti-melanoma differentiation-associated gene 5 (MDA5) antibody, and serum levels of C-reactive protein (CRP) and Krebs von den Lungen-6 (KL-6). The cluster model was further applied to 283 patients who received conventional regimens consisting of corticosteroids with or without a single immunosuppressive agent (dual-combo therapy or monotherapy). Cumulative survival rates were compared using Kaplan-Meier analysis, and the log-rank test was used to test for significant differences between two groups.

**Results:**

We developed a cluster model consisting of 6 clusters, which were categorized by age at onset, clinically amyopathic dermatomyositis, CRP, KL-6, requirement of supplemental oxygen, anti-ARS antibody, and anti-MDA5 antibody. This model was judged to be of good quality based on the silhouette measure of cohesion and separation of 0.6. These clusters were regrouped into three subsets based on low (<10%), moderate (10-50%), and high (>50%) mortality rates. The performance of the clustering was generally replicated in patients who received initial dual-combo therapy or monotherapy. Survival benefits of triple-combo therapy over dual-combo therapy or monotherapy were not observed in any of the clusters.

**Conclusion:**

We successfully developed a cluster model that stratified patients with myositis-associated ILD who were treated with initial triple-combo therapy into subgroups with different prognoses, although this model failed to identify a patient subgroup that showed survival benefits from triple-combo therapy over dual-combo therapy or monotherapy.

## Introduction

Idiopathic inflammatory myopathies often affect extramuscular organs such as the skin, joints, lungs, heart and gastrointestinal tract (1). In particular, interstitial lung disease (ILD) is one of the major manifestations associated with poor mortality in patients with polymyositis (PM)/dermatomyositis (DM) (2). The management of ILD in patients with PM/DM aims to ameliorate, stabilize, or slow its progression based on the disease behavior of ILD (3). In terms of treatment for myositis-associated ILD, systemic corticosteroid therapy is usually combined with immunosuppressive agents, such as azathioprine, cyclophosphamide, mycophenolate mofetil, methotrexate, cyclosporine, tacrolimus, and/or rituximab, although there is little evidence to support the efficacy of these individual agents (4). Since the clinical course, response to treatment, and prognosis are highly variable among patients with myositis-associated ILD (3, 5), the treatment regimen is decided based mainly on the progression speed and severity of ILD. On the other hand, many studies have reported clinical, laboratory, and imaging features predicting subsequent treatment response and prognosis (6–11).

Among myositis-specific autoantibodies, anti-melanoma differentiation-associated gene 5 (MDA5) and anti-aminoacyl tRNA synthetase (ARS) antibodies are associated with ILD (2). Anti-MDA5 antibody is useful for predicting rapidly progressive ILD and poor survival in patients with myositis-associated ILD (9, 12). Approximately 30 to 50% of patients with anti-MDA5-associated ILD die of respiratory failure within 6 months after diagnosis (9, 13, 14). In a large-scale multicenter retrospective cohort of Japanese patients with myositis-associated ILD (JAMI), the major cause of death was respiratory insufficiency directly related to ILD and ant-MDA5 antibody was the strongest predictor of mortality regardless of the initial treatment regimen (9, 11). To overcome this devastating condition, an intensive immunosuppressive regimen consisting of high-dose corticosteroids, a calcineurin inhibitor maintained at a high serum trough level, and intermittent intravenous cyclophosphamide (“triple-combo” therapy) is empirically used mainly in Japan without firm evidence for efficacy (15–17). A prospective, multicenter, single-arm study in patients with anti-MDA5-associated ILD suggested superiority of initial triple-combo therapy over historical controls who received sequential combination therapy (18). On the other hand, a retrospective cohort reported that more than half of patients with myositis-associated ILD who received triple-combo therapy developed serious infection events, including bacterial, fungal and cytomegalovirus infection (19). In addition, it has been reported that some patients with anti-MDA5-associated ILD favorably respond to corticosteroids combined with a single immunosuppressive agent (“dual-combo” therapy) or corticosteroids alone (monotherapy) (20). In fact, over 80% of patients with anti-MDA5 antibody survived without receiving initial triple-combo therapy in the JAMI cohort (our unpublished data). On the other hand, patients with myositis-associated ILD who were negative for anti-MDA5 antibody are sometimes resistant to initial dual-combo therapy (11); initial triple-combo therapy might have a role in this patient population. Therefore, a personalized approach is necessary to optimize the initial immunosuppressive regimen in patients with myositis-associated ILD, especially those with anti-MDA5-associated ILD. In this study, the JAMI cohort was used to stratify patients with myositis-associated ILD who were initially treated with triple-combo therapy into subgroups based on mortality outcomes by a cluster analysis.

## Materials and Methods

### Study Subjects

JAMI is a multicenter, retrospective cohort of 499 adult incident cases with myositis-associated ILD who visited 44 participating centers across Japan between October 2011 and October 2015. The detailed study protocol was described previously (9). Briefly, inclusion criteria were fulfillment of the criteria for definite or probable PM/DM proposed by Bohan and Peter (21) or Sontheimer's criteria for clinically amyopathic DM (CADM) (22), except that patients were not required to meet the condition of no clinical evidence of myositis for at least 6 months. Patients with ILD alone without any muscle involvement or hallmark cutaneous manifestation of DM were excluded. For this study, we selected 468 patients from the JAMI cohort based on a record of initiation of immunosuppressive treatment at diagnosis and availability of data required for the cluster analysis. Anti-ARS and anti-MDA5 antibodies were measured at the central laboratories with an RNA immunoprecipitation assay (23) and an in-house enzyme-linked immunosorbent assay (24), respectively. The treatment regimen was decided by attending physicians without information on autoantibodies. Triple-combo therapy was defined as a combined regimen consisting of high-dose corticosteroids, a calcineurin inhibitor, and intermittent intravenous cyclophosphamide, while dual-combo therapy was defined as a combination of corticosteroids and a single immunosuppressant, such as cyclophosphamide, cyclosporin, and tacrolimus. Monotherapy was defined as corticosteroids alone. Treatment drugs used within 2 weeks after treatment initiation were defined as an initial regimen. Before introduction of the immunosuppressive treatment, all patients underwent screening of latent infection of microorganisms, such as hepatitis B virus, *Pneumocystis jiroveci* and *Mycobacterium tuberculosis*, and prophylactic medications if necessary. The study protocol was approved by the Ethics Committee of the coordinating center (Nippon Medical School, Tokyo, Japan; 26-03-434) and by individual participating centers. The JAMI cohort was registered in the University Hospitals Medical Information Network Clinical Trial Registry (UMIN000018663).

### Statistical Analysis

All statistical analyses were conducted by an independent medical statistician (KM) using SPSS Statistics version 23 (IBM, Tokyo, Japan). Continuous values are shown as the median (25–75 percentile) according to the distribution of the data. Two-step cluster analysis of preclustering and subsequent hierarchical clustering was conducted in 185 patients who received triple-combo therapy in an unbiased manner. This procedure automatically determined the optimal number of clusters according to the Bayesian Information Criterion (25). Initial predictors for all-cause mortality or mortality due to respiratory insufficiency directly related to ILD reported in the JAMI cohort (9, 11), including age, sex, disease duration at diagnosis, classification of myositis, requirement of supplemental oxygen, anti-ARS antibody, anti-MDA5 antibody, and serum levels of C-reactive protein (CRP) and Krebs von den Lungen-6 (KL-6), were used as nominal and numerical variables. The number of clusters was estimated to organize homogeneous groups characterized by a combination of prognostic factors. We applied a final model that was divided into 3 groups stratified by mortality rates (low, moderate, and high) and comprised the greatest number of explanatory variables and clusters. In some instances, the two-step cluster analysis was conducted in anti-MDA5 antibody-positive patients alone; in this case, anti-MDA5 antibody was excluded from the initial predictor variables. The quality of the cluster model was assessed using the silhouette measure of cohesion and separation: ≥ 0.5 was shown to be good quality (25). The cluster model developed for patients receiving initial triple-combo therapy was also applied to patients who received dual-combo therapy or monotherapy using significant variables identified based on predictor importance, in which a score ≥ 0.4 indicated a significant variable (26).

To compare variables between the groups, the chi-square test, Fisher's exact test, or the Mann–Whitney U test was employed where applicable. Cumulative survival rates were compared using Kaplan–Meier analysis, and significant differences were tested using the log-rank test. *P* < 0.05 was considered statistically significant.

## Results

### Clinical Characteristics of Myositis-Associated ILD Patients Who Received Initial Triple-Combo Therapy

The JAMI cohort enrolled incident cases of myositis-associated ILD, with a short disease duration of 2 months (median) and a predominant disease classification of CADM (54%). Anti-ARS and anti-MDA5 antibodies were detected in 31% and 42% of patients, respectively. Of 468 patients, 185 (40%), 208 (44%), and 75 (16%) patients were initially treated with triple-combo therapy, dual-combo therapy, and monotherapy, respectively. The median follow-up period from the cohort entry to the latest visit or death was 19.5 (5–42) months. Table 1 shows the baseline characteristics of the 468 patients with myositis-associated ILD stratified by the initial treatment regimen. Clinical characteristics in patients who received triple-combo therapy in comparison with those who received dual-combo therapy or monotherapy included a higher prevalence of CADM, fever, skin ulcerations, lower consolidation/ground-glass attenuation and random ground-glass attenuation on chest high-resolution computed tomography, and requirement of supplemental oxygen; higher levels of CRP and ferritin; lower levels of CK and SP-D; and a higher proportion of anti-MDA5 antibody and lower proportion of anti-ARS antibody.

**Table 1:** Baseline characteristics of patients with myositis-ILD stratified by therapeutic regimen.

**Variables**	**Whole group** **(*n* = 468)**	**Available data** **per outcome**	**Triple-combo Tx** **(*n* = 185)**	**Dual-combo Tx** **(*n* = 208)**	**Mono Tx** **(*n* = 75)**	***P*** values*
Demographics						
Age at diagnosis, years	57 (47–65)	468 (100%)	59 (48–65)	51 (46–64)	67 (66–ND)	Triple Tx vs. Dual Tx: 0.04 Triple Tx vs. Mono Tx: 0.36 Dual Tx vs. Mono Tx: 0.02
Male, no. (%)	160 (34%)	468 (100%)	71 (38%)	61 (29%)	28 (37%)	Triple Tx vs. Dual Tx: 0.06 Triple Tx vs. Mono Tx: 0.88 Dual Tx vs. Mono Tx: 0.20
Disease duration at diagnosis, months	2 (1–5)	468 (100%)	2 (1–3)	3 (2–7)	2 (1–7)	Triple Tx vs. Dual Tx: 0.03 Triple Tx vs. Mono Tx: 0.44 Dual Tx vs. Mono Tx: 0.18
Disease classification						
PM, no. (%)	71 (15%)	468 (100%)	10 (5%)	47 (23%)	14 (19%)	Triple Tx vs. Dual Tx: 0.01 Triple Tx vs. Mono Tx: <0.001 Dual Tx vs. Mono Tx: 0.74
Classic DM, no. (%)	144 (31%)		42 (23%)	73 (35%)	29 (39%)	
CADM, no. (%)	253 (54%)		133 (72%)	88 (42%)	32 (43%)	
Clinical features						
Fever, no. (%)	223 (49%)	455 (97%)	121 (65%)	85 (42%)	17 (26%)	Triple Tx vs. Dual Tx: <0.001 Triple Tx vs. Mono Tx: <0.001 Dual Tx vs. Mono Tx: 0.02
Raynaud's phenomenon, no. (%)	63 (15%)	419 (90%)	12 (8%)	40 (20%)	11 (17%)	Triple Tx vs. Dual Tx: <0.001 Triple Tx vs. Mono Tx: 0.32 Dual Tx vs. Mono Tx: 0.57
Arthritis/arthralgia, no. (%)	213 (46%)	445 (95%)	91 (51%)	99 (50%)	23 (34%)	Triple Tx vs. Dual Tx: 0.83 Triple Tx vs. Mono Tx: 0.02 Dual Tx vs. Mono Tx: 0.03
Skin ulceration, no. (%)	44 (9%)	432 (92%)	28 (16%)	12 (6%)	4 (7%)	Triple Tx vs. Dual Tx: 0.002 Triple Tx vs. Mono Tx: 0.07 Dual Tx vs. Mono Tx: 0.87
Laboratory parameters						
CK, IU/L	199 (78–748)	460 (98%)	159 (76–439)	206 (80–1,298)	312 (99–1,200)	Triple Tx vs. Dual Tx: 0.10 Triple Tx vs. Mono Tx: 0.05 Dual Tx vs. Mono Tx: 0.41
Aldolase, IU/L	9.0 (6.7–18.6)	400 (85%)	8.2 (6.4–12.9)	10.6 (6.9–22.8)	8.8 (7.1–21.7)	Triple Tx vs. Dual Tx: 0.65 Triple Tx vs. Mono Tx: 0.38 Dual Tx vs. Mono Tx: 0.89
CRP, mg/dL	0.8 (0.2–2.1)	453 (99%)	1.1 (0.3–2.5)	0.6 (0.2–1.8)	0.5 (0.1–1.9)	Triple Tx vs. Dual Tx: <0.001 Triple Tx vs. Mono Tx: 0.002 Dual Tx vs. Mono Tx: 0.86
Ferritin, ng/mL	353 (141–767)	344 (75%)	645 (267–1,213)	251 (117–573)	212 (115–373)	Triple Tx vs. Dual Tx <0.001 Triple Tx vs. Mono Tx <0.001 Dual Tx vs. Mono Tx 0.05
KL-6, U/mL	803 (540-1,268)	454 (97%)	762 (541–1,226)	865 (567–1,428)	716 (459–1,101)	Triple Tx vs. Dual Tx: 0.17 Triple Tx vs. Mono Tx: 0.10 Dual Tx vs. Mono Tx: 0.008
SP-D, ng/mL	94 (48–176)	356 (76%)	64 (37–134)	116 (62–215)	137 (93–242)	Triple Tx vs. Dual Tx: <0.001 Triple Tx vs. Mono Tx: <0.001 Dual Tx vs. Mono Tx: 0.18
Chest HRCT findings						
Lower consolidation/GGA, no. (%)	257 (55%)	467 (99%)	120 (65%)	104 (50%)	33 (44%)	Triple Tx vs. Dual Tx: 0.002 Triple Tx vs. Mono Tx: 0.002 Dual Tx vs. Mono Tx: 0.37
Lower reticulation, no. (%)	152 (33%)	467 (99%)	37 (20%)	81 (39%)	34 (45%)	Triple Tx vs. Dual Tx: <0.001 Triple Tx vs. Mono Tx: <0.001 Dual Tx vs. Mono Tx: 0.33
Random GGA, no. (%)	57 (12%)	467 (99%)	32 (17%)	19 (9%)	6 (8%)	Triple Tx vs. Dual Tx: 0.02 Triple Tx vs. Mono Tx: 0.05 Dual Tx vs. Mono Tx: 0.77
Supplemental oxygen, no. (%)	46 (10%)	468 (100%)	33 (18%)	11 (5%)	2 (3%)	Triple Tx vs. Dual Tx: <0.001 Triple Tx vs. Mono Tx: <0.001 Dual Tx vs. Mono Tx: 0.35
Myositis-specific autoantibodies**
Anti-ARS antibody, no. (%)	155 (31%)	468 (100%)	24 (13%)	93 (45%)	38 (51%)	Triple Tx vs. Dual Tx: <0.001 Triple Tx vs. Mono Tx: <0.001 Dual Tx vs. Mono Tx: 0.78
Anti-MDA5 antibody, no. (%)	195 (42%)	468 (100%)	133 (72%)	54 (26%)	8 (11%)	Triple Tx vs. Dual Tx: <0.001 Triple Tx vs. Mono Tx: <0.001 Dual Tx vs. Mono Tx: 0.006

*Continuous variables are shown as the median (25–75 percentile)*.

* *Comparisons between two groups using the chi-square test, Fisher's exact test or Mann–Whitney U test when applicable*.

** *Two patients had both anti-ARS and anti-MDA5 antibodies*.

*ILD, interstitial lung disease; Tx, therapy; ND, not determinate; PM, polymyositis; DM, dermatomyositis; CADM, clinically amyopathic dermatomyositis; CK, creatine kinase; CRP, C-reactive protein; KL-6, Krebs von den Lungen-6; SP-D, surfactant protein-D; HRCT, high-resolution computed tomography; GGA, ground-glass attenuation; anti-ARS, anti-aminoacyl tRNA synthetase; anti-MDA5, anti-melanoma differentiation-associated gene 5*.

### Clustering of Myositis-Associated ILD Patients Who Received Initial Triple-Combo Therapy Based on Mortality Rates

**Figure 1:**
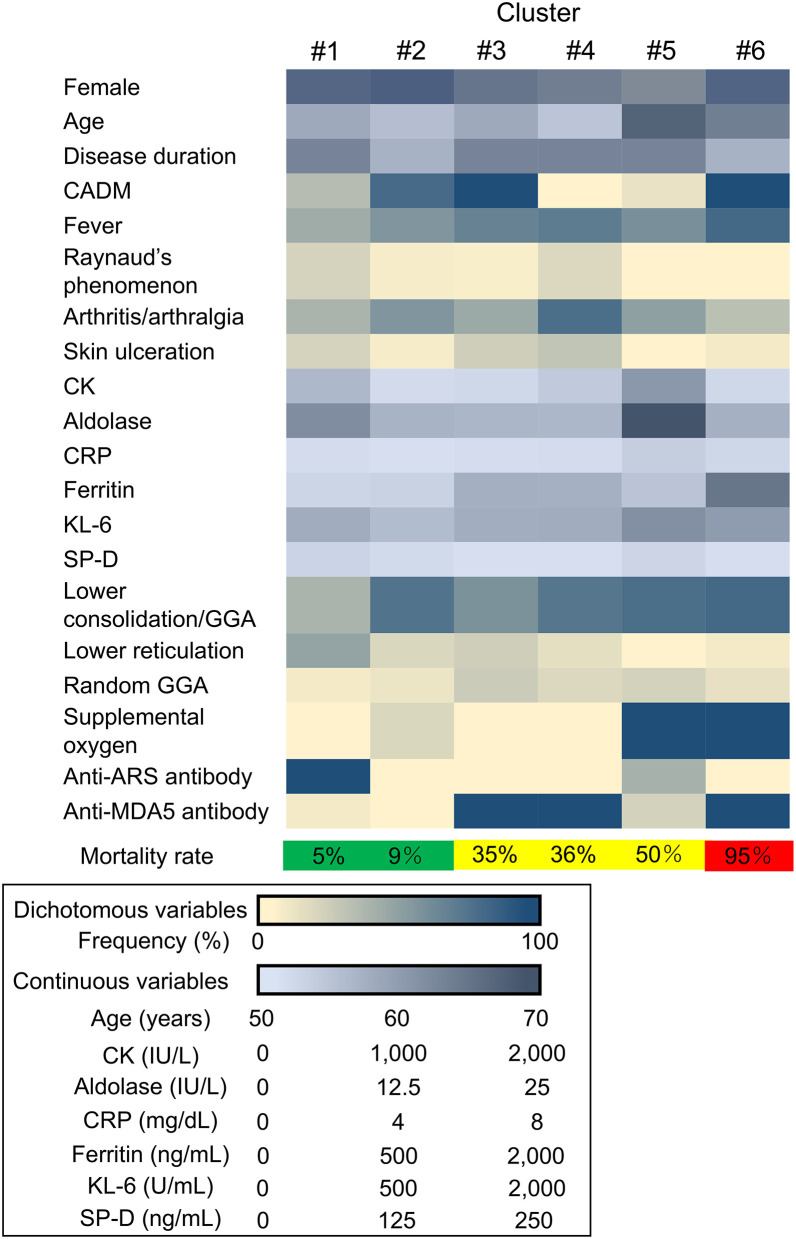
Heatmap showing the clinical characteristics in each cluster. CADM, clinically amyopathic dermatomyositis; CK, creatine kinase; CRP, C-reactive protein; KL-6, Krebs von den Lungen-6; SP-D, surfactant protein-D; GGA, ground-glass attenuation; anti-ARS, anti-aminoacyl transfer RNA synthetase; anti-MDA5, anti-melanoma differentiation-associated gene 5.

The unbiased two-step cluster analysis in 185 patients with myositis-associated ILD who received initial triple-combo therapy identified 6 clusters, which were categorized by the following 7 explanatory variables: age at disease onset, CADM, CRP, KL-6, requirement of supplemental oxygen, anti-ARS antibody, and anti-MDA5 antibody (Figure 1). The silhouette measure of cohesion and separation of this clustering model was 0.6, indicating good quality. These clusters were regrouped into three groups based on low mortality rate (<10%; clusters #1 and #2), moderate mortality rate (10–50%; clusters #3, #4, and #5), and high mortality rate (>50%; cluster #6).

**Figure 2:**
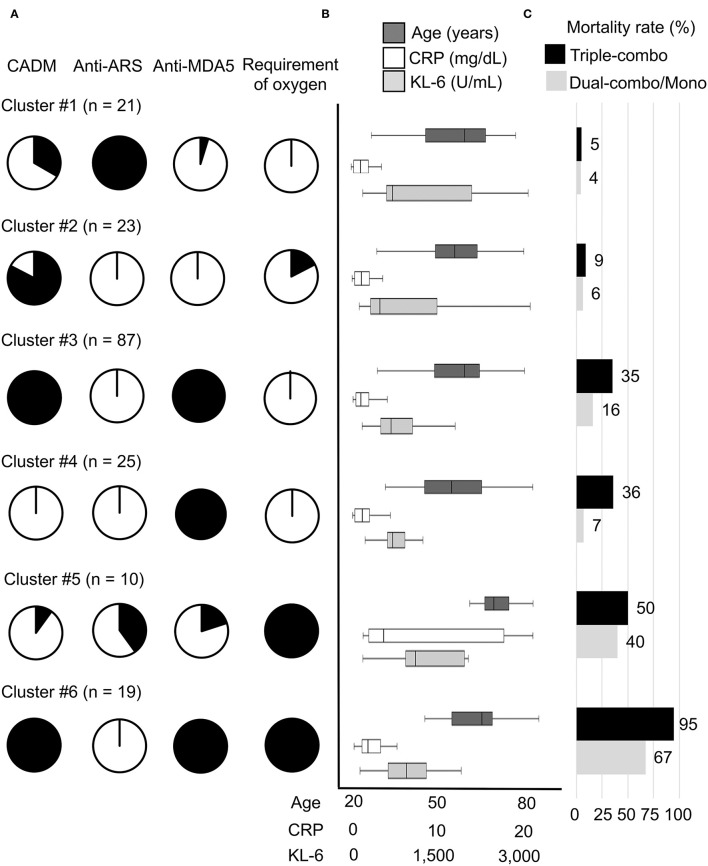
Main characteristics of the 6 clusters (clusters #1–#6) of patients with myositis-associated ILD treated with initial triple-combo therapy. **(A)** Proportions of each cluster with the main clinical characteristics of clinically amyopathic dermatomyositis (CADM), anti-aminoacyl transfer RNA synthetase (ARS) antibody, anti-melanoma differentiation-associated gene 5 (MDA5) antibody, and requirement of supplemental oxygen. **(B)** Age at disease onset and serum levels of C-reactive protein (CRP) and Krebs von den Lungen-6 (KL-6) at diagnosis in each cluster. **(C)** Mortality rates during the observation period in patients treated with initial triple-combo therapy and those treated with dual-combo therapy or monotherapy in each cluster.

**Figure 3:**
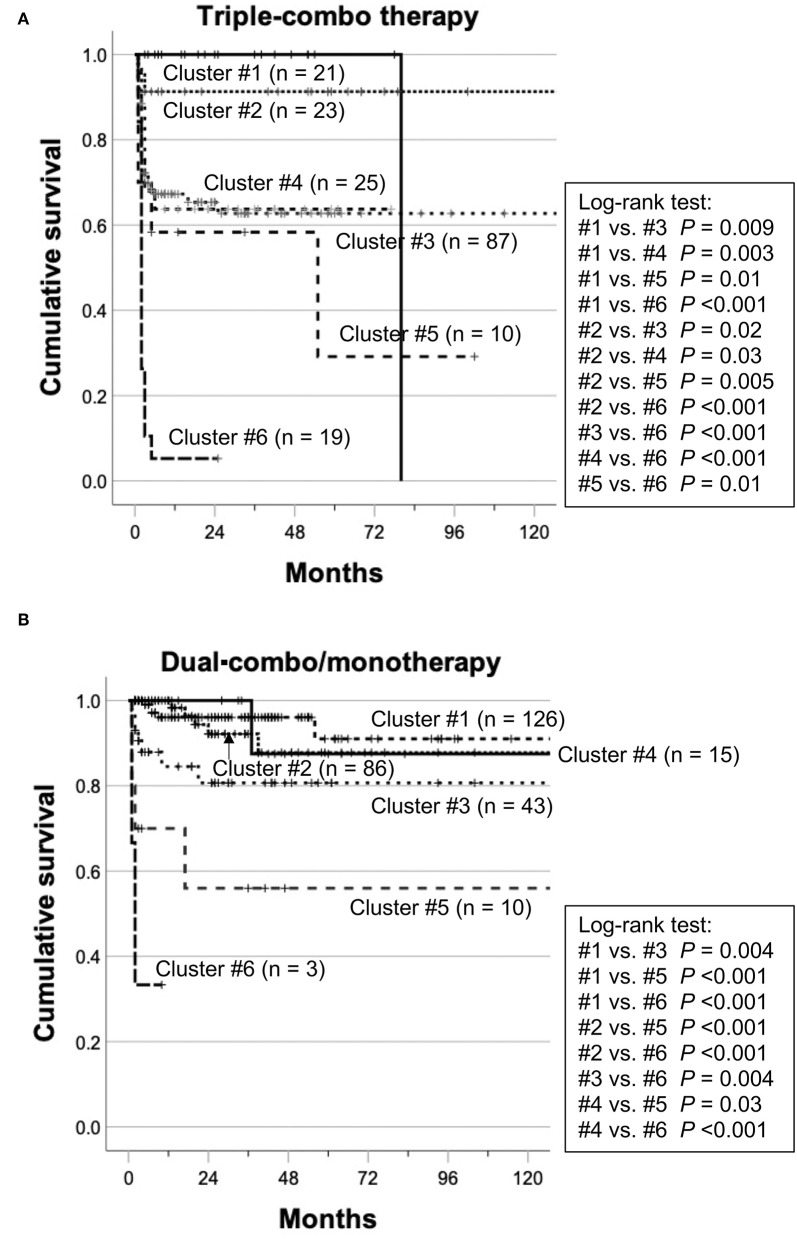
Cumulative survival rates in each cluster in patients treated with initial triple-combo therapy **(A)** or those treated with initial dual-combo therapy or monotherapy **(B)**. Cumulative survival rates were compared using Kaplan–Meier analysis, and the log-rank test was used to test for significant differences between two groups.

The patients with myositis-associated ILD who initially received triple-combo therapy were divided into 6 clusters, and clinical characteristics in individual clusters are shown in Supplementary Table S1. The median age at disease onset was older in cluster #5. CADM was more common in clusters #2, #3, and #6. In terms of autoantibody profiles, anti-ARS antibody was more frequent in cluster #1, while anti-MDA5 antibody was more frequent in clusters #3, #4, and #6. Supplemental oxygen was frequently required in clusters #5 and #6. The levels of serum CRP and KL-6 were higher in clusters #5 and #6. The overall patient profiles at presentation in individual clusters are illustrated in Figure 2. These include cluster #1, anti-ARS antibody-positive patients without the requirement of supplemental oxygen; cluster #2; patients negative for anti-ARS or anti-MDA5 antibody without the requirement of supplemental oxygen; cluster #3, anti-MDA5 antibody-positive patients classified as having CADM without the requirement of supplemental oxygen; cluster #4, anti-MDA5 antibody-positive patients classified as having classic DM without the requirement of supplemental oxygen; cluster #5, anti-MDA5 antibody-negative elderly patients with high levels of CRP and KL-6 who required supplemental oxygen; and cluster #6, anti-MDA5 antibody-positive patients classified as having CADM who required supplemental oxygen. Kaplan–Meier analysis demonstrated that cumulative survival rates were significantly different between patients in clusters #1 or #2 (low mortality group) and those in clusters #3, #4, or #5 (moderate mortality group) or #6 (high mortality group) or between patients in clusters #3, #4 or #5 (moderate mortality group) and those in cluster #6 (high mortality group) (Figure 3A).

Since anti-MDA5 antibody was the strongest predictor of mortality in the JAMI cohort (9), we further conducted the two-step cluster analysis in 134 patients with anti-MDA5 antibody who initially received triple-combo therapy (Supplementary Table S2). The patients were divided into three clusters, but separation based on mortality rate was not efficient; clusters were regrouped into two groups based on moderate mortality rate (10–50%; clusters #M1 and #M2), and high mortality rate (>50%; cluster #M3). In fact, the clusters derived from the analysis of anti-MDA5-positive patients were almost concordant to those from the analysis of the whole cohort. Specifically, the patients included in cluster #M1 and cluster #3 were identical, while the patients included in clusters #M2 and #M3 were almost the same as those included in clusters #4 and #6, with one or two additions, respectively.

### Application of the Cluster Model to Myositis-Associated ILD Patients Who Received Initial Dual-Combo Therapy or Monotherapy

We further tested whether the clustering model developed for patients who received initial triple-combo therapy was applicable to patients who received initial dual-combo therapy or monotherapy. Anti-MDA5 antibody, anti-ARS antibody, CADM, and requirement of supplemental oxygen were identified as significant variables for application in the clustering model for patients who received initial dual-combo therapy or monotherapy based on predictor importance (Supplementary Figure S1). When a total of 283 patients who received initial dual-combo therapy or monotherapy were combined, the performance of the clustering was generally replicated in this patient population: mortality rates in clusters #1–#6 were 4, 6, 16, 7, 40, and 67%, respectively, while there was a considerable difference in the mortality rate between patients in clusters #3 and #4 and those in cluster #5 (Figure 2). The clinical characteristics of patients in each cluster were somewhat different between the triple-combo and dual-combo therapy or monotherapy groups, i.e., a low prevalence of CADM and a high prevalence of the requirement of supplemental oxygen in cluster #2, a younger age at diagnosis and a lower level of CRP in cluster #3, and a lower level of CRP in cluster #4 (Supplementary Table S3). These clusters fell into two groups, clusters #1-#4 and clusters #5 and #6, based on cumulative survival rates (Figure 3B).

**Figure 4:**
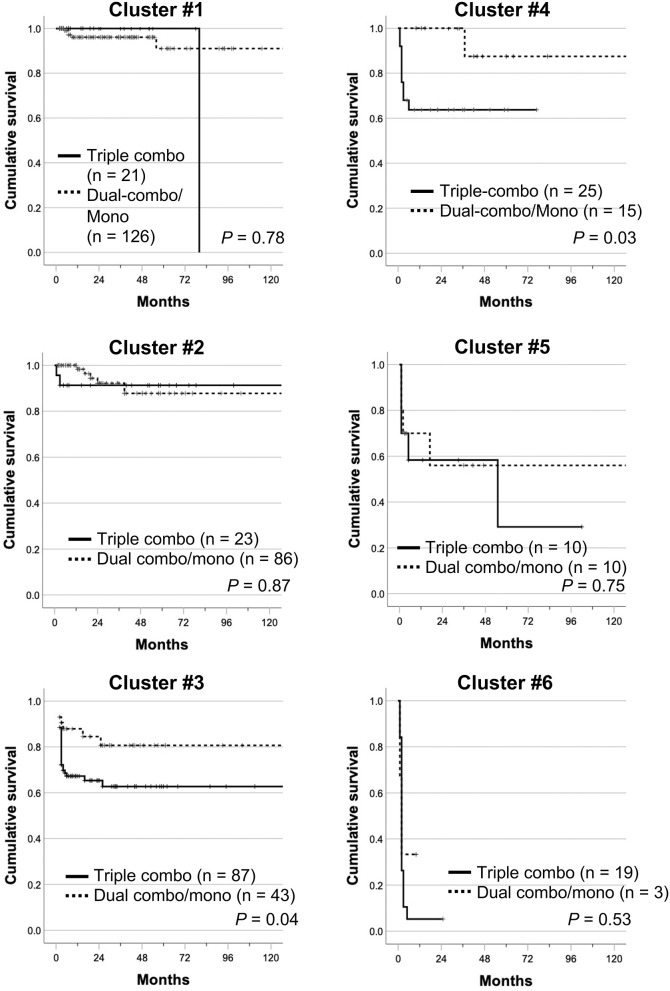
Cumulative survival rates of patients treated with initial triple-combo therapy and those treated with initial dual-combo therapy or monotherapy in each cluster (clusters #1–#6). Cumulative survival rates were compared using Kaplan–Meier analysis, and the log-rank test was used to test for significant differences between two groups.

When cumulative survival rates in each cluster were compared between the triple-combo therapy and dual-combo therapy or monotherapy groups, there was no cluster that showed survival benefits from triple-combo therapy over dual-combo therapy or monotherapy groups (Figure 4). In addition, cumulative survival rates were even lower in the triple-combo therapy group than in the dual-combo therapy or monotherapy group in clusters #3 and #4 (*P* = 0.04 and 0.03, respectively).

## Discussion

We successfully developed a clustering model that predicts survival in patients with myositis-associated ILD who received initial triple-combo therapy. The patient subsets categorized by optimal clustering differed with regard to age at disease onset, disease classification, autoantibodies, serum biomarkers, and severity of ILD, which are known as predicting factors for mortality in patients with myositis-associated ILD (6–11). Our findings suggest that subgrouping of patients with myositis-associated ILD based solely on the presence or absence of anti-MDA5 antibody may not capture the heterogeneous treatment responses to the intensive immunosuppressive regimen. This model enables us to predict the survival of patients with myositis-associated ILD undergoing triple-combo therapy but fails to identify patients who benefit from initial triple-combo therapy over dual-combo therapy or monotherapy. It was of note that prognosis in patients with myositis-associated ILD correlated with baseline characteristics, such as autoantibodies profiles and ILD severity, rather than the intensity of initial immunosuppressive treatment.

Triple-combo therapy has often been used for myositis patients who develop acute/subacute ILD, particularly those with anti-MDA5 antibody (15–17). Survival rates in patients with anti-MDA5-associated ILD who received triple-combo therapy ranged from 42 to 85% according to previous reports (16, 18, 27, 28). Our cluster model was able to classify anti-MDA5-positive patients who received triple-combo therapy primarily into two subsets based on prognosis: a subset with moderate mortality rates (clusters #3 and #4) and a subset with high mortality rates (cluster #6). In addition, a minor population of anti-MDA5-positive patients was included in the low mortality group (cluster #1). Cluster #6, with poor treatment responses to triple-combo therapy, was characterized by older age at disease onset, increased levels of CRP and KL-6, and hypoxia at diagnosis, which were reported as poor prognostic factors in patients with myositis-associated ILD (9, 11). These patients probably require novel treatment approaches, such as the use of a Janus kinase inhibitor and plasma exchange (29–36). However, no survival benefit of triple-combo therapy over dual-combo therapy or monotherapy was shown in clusters #3, #4, or #6. In addition, our cluster model failed to identify patients with anti-MDA5-associated ILD who did not require triple-combo therapy. Finally, our findings did not support a better prognosis in patients with anti-MDA5-associated ILD treated with initial triple-combo therapy over those treated initially with dual-combo therapy or monotherapy (18). This finding was a rather unexpected. The precise reason for considerable difference in the mortality between anti-MDA5 antibody-positive patients undergoing triple-combo therapy in the study by Tsuji et al. (18) and those in the JAMI cohort was unclear, but this might be attributable to the different criteria for inclusion. In the study by Tsuji et al. (18), all consecutive patients identified to be positive for anti-MDA5 antibody received initial triple-combo therapy irrespective of the prognostic factors. On the other hand, in the JAMI cohort, anti-MDA5 antibody was measured using the stored serum samples later in the central laboratories and attending physicians had to decide initial treatment regimen without knowing the presence or absence of anti-MDA5 antibody. Therefore, in the JAMI cohort, triple combo therapy was chosen based on physician's expertise, not on information of the presence of anti-MDA5 antibody. Actually, 28% of the patients in the triple-combo therapy group were anti-MDA5 antibody-negative. The patients with more severe ILD or those with multiple prognostic factors for poor outcomes were likely to be included in the triple-combo therapy group in the JAMI cohort.

On the other hand, the efficacy of triple-combo therapy in patients negative for anti-MDA5 antibody remains uncertain. In our clustering model, clusters #1, #2, and #5 mainly consisted of anti-MDA5 antibody-negative patients. According to previous reports on anti-MDA5 antibody-negative patients with myositis-associated ILD, the cumulative survival rate over 5 years was >80%, regardless of the initial immunosuppressive regimen (12, 37–39). Our cluster model identified patients who had worse survival despite treatment with triple-combo therapy among anti-MDA5 antibody-negative patients with myositis-associated ILD (cluster #5). The cluster #5 consisted mainly of elderly patients with high levels of CRP and KL-6 who required supplemental oxygen at diagnosis. Patients with such features at presentation need to be managed with caution even in the absence of anti-MDA5 antibody. On the other hand, in anti-MDA5 antibody-negative patients categorized into clusters #1 and #2, there was no survival benefit of triple-combo therapy over dual-combo therapy or monotherapy.

Unexpectedly, in clusters #3 and #4, survival rates were better in the dual-combo therapy or monotherapy group than in the triple-combo therapy group. This finding needs to be carefully interpreted, and could be potentially due to inadequate adaptation of the clustering model developed for patients treated with triple-combo therapy to those treated with dual-combo therapy or monotherapy. In fact, clinical characteristics were somewhat different between the triple-combo therapy group and the dual-combo therapy or monotherapy group in individual clusters. Since the treatment regimen was decided by attending physicians based on their expertise without information of autoantibody profiles in the JAMI cohort, the JAMI dataset might not be able to capture all relevant factors related to the physician's expertise for selection of the initial treatment regimen, such as patient report outcomes and physician's global estimate of patient status.

There are several limitations in this study. First, the participating centers of the JAMI cohort consist mainly of tertiary referral hospitals, which are likely to enroll patients with more severe disease, such as those with anti-MDA5 antibody. Second, the JAMI cohort did not enroll patients with anti-ARS antibody without any muscle or skin symptoms, which are often encountered in clinical practice. Third, pulmonary function test and arterial blood gas data at diagnosis were missing in considerable proportions of patients enrolled in the JAMI cohort, while baseline forced vital capacity was identified as the prognostic factor for mortality in anti-MDA5 antibody-positive patients with DM-associated ILD (40). Finally, we used initial predictors for all-cause mortality or mortality due to respiratory insufficiency directly related to ILD reported in previous reports using the JAMI cohort (9, 11) as variables in the cluster analysis, although there are many other prognostic factors for mortality in patients with myositis-associated ILD reported previously (6–8, 10, 39, 40).

## Conclusion

We successfully developed a cluster model that categorizes patients with myositis-associated ILD who were treated with initial triple-combo therapy into subgroups with different prognoses. However, the patient subgroup that benefitted from triple-combo therapy was not identified in this cluster analysis.

## Data Availability Statement

The raw data supporting the conclusions of this article will be made available by the authors, without undue reservation.

## Ethics Statement

The studies involving human participants were reviewed and approved by Ethics Committee of Nippon Medical School. The patients/participants provided their written informed consent to participate in this study.

## Multicenter Retrospective Cohort of Japanese Patients With Myositis-Associated ILD (Jami) Investigators

Naoshi Nishina and Yuko Kaneko, Keio University School of Medicine, Tokyo, Japan; Yasushi Kawaguchi, Tokyo Women's Medical University, Tokyo, Japan; Atsushi Kawakami, Nagasaki University Graduate School of Biomedical Sciences, Nagasaki, Japan; Kei Ikeda, Chiba University Hospital, Chiba, Japan; Yohei Kirino and Yukie Yamaguchi, Yokohama City University Graduate School of Medicine, Yokohama, Japan; Yumiko Sugiyama, Yokohama City University Medical Center, Yokohama, Japan; Yoshinori Tanino, Fukushima Medical University School of Medicine, Fukushima, Japan; Takahiro Nunokawa, Tokyo Metropolitan Tama Medical Center, Fuchu, Japan; Katsuaki Asakawa, Niigata University Medical and Dental Hospital, Niigata, Japan; Taro Ukichi, The Jikei University School of Medicine, Tokyo, Japan; Shinjiro Kaieda, Kurume University School of Medicine, Kurume, Japan; Taio Naniwa, Nagoya City University School of Medicine, Nagoya, Japan; Yutaka Okano, Kawasaki Municipal Hospital, Kawasaki, Japan; Yoshinori Taniguchi, Kochi Medical School, Kochi University, Kochi, Japan; Jun Kikuchi, Saitama Medical Center, Saitama Medical University, Saitama, Japan; Makoto Kubo, Yamaguchi University Graduate School of Medicine, Yamaguchi, Japan, Masaki Watanabe, Graduate School of Medical and Dental Sciences, Kagoshima University, Kagoshima, Japan; Tatsuhiko Harada, Nagasaki University School of Medicine, Nagasaki, Japan; Taisuke Kazuyori, The Jikei University School of Medicine Katsushika Medical Center, Tokyo, Japan; Hideto Kameda, Toho University Ohashi Medical Center, Tokyo, Japan; Makoto Kaburaki, Toho University School of Medicine, Tokyo, Japan; Yasuo Matsuzawa, Toho University Medical Center, Sakura Hospital, Tokyo, Japan; Shunji Yoshida, Fujita Health University School of Medicine, Toyoake, Japan; Yasuko Yoshioka and Takuya Hirai, Juntendo University Urayasu Hospital, Chiba, Japan; Yoko Wada, Niigata University Graduate School of Medical and Dental Sciences, Niigata, Japan; Koji Ishii, Faculty of Medicine, Oita University, Oita, Japan; Sakuhei Fujiwara, Faculty of Medicine Oita University, Oita, Japan; Takeshi Saraya, Kyorin University, Mitaka, Japan; Kozo Morimoto, Fukujuji Hospital, Japan Anti-Tuberculosis Association, Kiyose, Japan; Tetsu Hara, Hiratsuka Kyosai Hospital, Hiratsuka, Japan; Hiroki Suzuki, Saiseikai Yamagata Saisei Hospital, Yamagata, Japan; Hideki Shibuya, Tokyo Teishin Hospital, Tokyo, Japan; Yoshinao Muro, Nagoya University Graduate School of Medicine, Nagoya, Japan; Ryoichi Aki, Kitasato University School of Medicine, Sagamihara, Japan; Takuo Shibayama, National Hospital Organization Okayama Medical Center, Okayama, Japan; Shiro Ohshima, National Hospital Organization Osaka Minami Medical Center, Kawachinagano, Japan; Yuko Yasuda, Saiseikai Kumamoto Hospital, Kumamoto, Japan; Masaki Terada, Saiseikai Niigata Daini Hospital, Niigata, Japan; Yoshie Kawahara, Keiyu Hospital, Yokohama, Japan.

## Author Contributions

TG, KM, SS, and MK: study conception and design. TG, KM, SS, MK, and JAMI Investigators: acquisition of data. TG and KM: analysis and interpretation of data. All authors draft the article or revise it critically for important intellectual content. All authors contributed to the article and approved the submitted version.

## Funding

This work was supported in part by a research grant for intractable diseases from the Japanese Ministry of Health, Labour and Welfare (20FC1050), Japan Agency for Medical Research and Development (21ek0109531h0001), and a research grant from Astellas. The funders had no role in the study design, data collection and analysis, decision to publish, or preparation of the manuscript.

## Conflict of Interest

TG received speaking fees from Astellas, Boehringer Ingelheim, Bristol-Myers Squibb, Janssen, MBL, Nippon Shinyaku, and Ono Pharmaceuticals. SS holds the patent for the anti-MDA5 antibody measurement kit. MK holds the patent for the anti-MDA5 antibody measurement kit and received consulting fees, speaking fees, and research grants from AbbVie, Actelion, Asahi Kasei, Astellas, Boehringer Ingelheim, Bayer, Chugai, Eisai, Corbus, Janssen, Kissei, MBL, Mitsubishi Tanabe, Mochida, Nippon Shinyaku, Pfizer, Ono Pharmaceuticals, Reata, and Teijin. The remaining author declares that the research was conducted in the absence of any commercial or financial relationships that could be construed as a potential conflict of interest.

## Publisher's Note

All claims expressed in this article are solely those of the authors and do not necessarily represent those of their affiliated organizations, or those of the publisher, the editors and the reviewers. Any product that may be evaluated in this article, or claim that may be made by its manufacturer, is not guaranteed or endorsed by the publisher.

## References

[1] Lundberg IE Fujimoto M Vencovsky J Aggarwal R Holmqvist M Christopher-Stine L. Idiopathic inflammatory myopathies. Nat Rev Dis Primers. (2021) 7:86. 10.1038/s41572-021-00321-x 34857798

[2] Gono T Kuwana M. Inflammatory myopathies: choosing the right biomarkers to predict ILD in myositis. Nat Rev Rheumatol. (2016) 12:504–6. 10.1038/nrrheum.2016.120 27440427

[3] Kondoh Y Makino S Ogura T Suda T Tomioka H Amano H. 2020 guide for the diagnosis and treatment of interstitial lung disease associated with connective tissue disease. Respir Investig. (2021) 59:709–40. 10.1016/j.resinv.2021.04.011 34602377

[4] Barsotti S Lundberg IE. Current treatment for myositis. Curr Treatm Opt Rheumatol. (2018) 4:299–315. 10.1007/s40674-018-0106-2 30613465 PMC6299051

[5] Kawasumi H Gono T Kawaguchi Y Yamanaka H. Recent treatment of interstitial lung disease with idiopathic inflammatory myopathies. Clin Med Insights Circ Respir Pulm Med. (2015) 9:9–17. 10.4137/CCRPM.S23313 26279636 PMC4514184

[6] Fujisawa T Hozumi H Kono M Enomoto N Hashimoto D Nakamura Y. Prognostic factors for myositis-associated interstitial lung disease. PLoS One. (2014) 9:e98824. 10.1371/journal.pone.0098824 24905449 PMC4048238

[7] Fujisawa T Hozumi H Kono M Enomoto N Nakamura Y Inui N. Predictive factors for long-term outcome in polymyositis/dermatomyositis-associated interstitial lung diseases. Respir Investig. (2017) 55:130–7. 10.1016/j.resinv.2016.09.006 28274528

[8] Isoda K Takeuchi T Kotani T Hata K Shoda T Ishida T. Pre-treatment ferritin level and alveolar-arterial oxygen gradient can predict mortality rate due to acute/subacute interstitial pneumonia in dermatomyositis treated by cyclosporine a/glucocorticosteroid combination therapy: a case control study [corrected]. PLoS ONE. (2014) 9:e89610. 10.1371/journal.pone.0089610 24586910 PMC3931830

[9] Sato S Masui K Nishina N Kawaguchi Y Kawakami A Tamura M. Initial predictors of poor survival in myositis-associated interstitial lung disease: a multicentre cohort of 497 patients. Rheumatology (Oxford). (2018) 57:1212–21. 10.1093/rheumatology/key060 29596687

[10] Tanizawa K Handa T Nakashima R Kubo T Hosono Y Aihara K. The prognostic value of HRCT in myositis-associated interstitial lung disease. Respir Med. (2013) 107:745–52. 10.1016/j.rmed.2013.01.014 23485097

[11] Gono T Masui K Nishina N Kawaguchi Y Kawakami A Ikeda K. Risk Prediction modeling based on a combination of initial serum biomarker levels in polymyositis/dermatomyositis-associated interstitial lung disease. Arthritis Rheumatol. (2021) 73:677–86. 10.1002/art.41566 33118321

[12] Moghadam-Kia S Oddis CV Sato S Kuwana M Aggarwal R. Anti-melanoma differentiation-associated gene 5 is associated with rapidly progressive lung disease and poor survival in US patients with amyopathic and myopathic dermatomyositis. Arthritis Care Res (Hoboken). (2016) 68:689–94. 10.1002/acr.22728 26414240 PMC4864500

[13] Nakashima R Imura Y Kobayashi S Yukawa N Yoshifuji H Nojima T. The RIG-I-like receptor IFIH1/MDA5 is a dermatomyositis-specific autoantigen identified by the anti-CADM-140 antibody. Rheumatology (Oxford). (2010) 49:433–40. 10.1093/rheumatology/kep375 20015976

[14] Koga T Fujikawa K Horai Y Okada A Kawashiri SY Iwamoto N. The diagnostic utility of anti-melanoma differentiation-associated gene 5 antibody testing for predicting the prognosis of Japanese patients with DM. Rheumatology (Oxford). (2012) 51:1278–84. 10.1093/rheumatology/ker518 22378718

[15] Kameda H Nagasawa H Ogawa H Sekiguchi N Takei H Tokuhira M. Combination therapy with corticosteroids, cyclosporin A, and intravenous pulse cyclophosphamide for acute/subacute interstitial pneumonia in patients with dermatomyositis. J Rheumatol. (2005) 32:1719–26. 16142867

[16] Nakashima R Hosono Y Mimori T. Clinical significance and new detection system of autoantibodies in myositis with interstitial lung disease. Lupus. (2016) 25:925–33. 10.1177/0961203316651748 27252271

[17] Romero-Bueno F Diaz Del Campo P Trallero-Araguás E Ruiz-Rodríguez JC Castellvi I Rodriguez-Nieto MJ. Recommendations for the treatment of anti-melanoma differentiation-associated gene 5-positive dermatomyositis-associated rapidly progressive interstitial lung disease. Semin Arthritis Rheum. (2020) 50:776–90. 10.1016/j.semarthrit.2020.03.007 32534273 PMC11616672

[18] Tsuji H Nakashima R Hosono Y Imura Y Yagita M Yoshifuji H. Multicenter prospective study of the efficacy and safety of combined immunosuppressive therapy with high-dose glucocorticoid, tacrolimus, and cyclophosphamide in interstitial lung diseases accompanied by anti-melanoma differentiation-associated gene 5-positive dermatomyositis. Arthritis Rheumatol. (2020) 72:488–98. 10.1002/art.41105 31524333

[19] Sugiyama Y Yoshimi R Tamura M Takeno M Kunishita Y Kishimoto D. The predictive prognostic factors for polymyositis/dermatomyositis-associated interstitial lung disease. Arthritis Res Ther. (2018) 20:7. 10.1186/s13075-017-1506-7 29325580 PMC5765702

[20] Waseda Y. Myositis-related interstitial lung disease: a respiratory physician's point of view. Medicina (Kaunas). (2021) 57:599. 10.3390/medicina57060599 34200737 PMC8230365

[21] Bohan A Peter JB. Polymyositis and dermatomyositis (first of two parts). N Engl J Med. (1975) 292:344–7. 10.1056/NEJM197502132920706 1090839

[22] Gerami P Schope JM McDonald L Walling HW Sontheimer RD. A systematic review of adult-onset clinically amyopathic dermatomyositis (dermatomyositis siné myositis): a missing link within the spectrum of the idiopathic inflammatory myopathies. J Am Acad Dermatol. (2006) 54:597–613. 10.1016/j.jaad.2005.10.041 16546580

[23] Forman MS Nakamura M Mimori T Gelpi C Hardin JA. Detection of antibodies to small nuclear ribonucleoproteins and small cytoplasmic ribonucleoproteins using unlabeled cell extracts. Arthritis Rheum. (1985) 28:1356–61. 10.1002/art.1780281207 2935157

[24] Sato S Hoshino K Satoh T Fujita T Kawakami Y Kuwana M. Helicase encoded by melanoma differentiation-associated gene 5 is a major autoantigen in patients with clinically amyopathic dermatomyositis: association with rapidly progressive interstitial lung disease. Arthritis Rheum. (2009) 60:2193–200. 10.1002/art.24621 19565506

[25] Wendler T Gröttru S. Cluster Analysis. Cham: Springer (2016). 10.1007/978-3-319-28709-6_7

[26] Martić-Kehl MI Wernery J Folkers G Schubiger PA. Quality of animal experiments in anti-angiogenic cancer drug development–a systematic review. PLoS ONE. (2015) 10:e0137235. 10.1371/journal.pone.0137235 26421849 PMC4589433

[27] Gono T Sato S Kawaguchi Y Kuwana M Hanaoka M Katsumata Y. Anti-MDA5 antibody, ferritin and IL-18 are useful for the evaluation of response to treatment in interstitial lung disease with anti-MDA5 antibody-positive dermatomyositis. Rheumatology (Oxford). (2012) 51:1563–70. 10.1093/rheumatology/kes102 22589330

[28] Muro Y Sugiura K Akiyama M. Limitations of a single-point evaluation of anti-MDA5 antibody, ferritin, and IL-18 in predicting the prognosis of interstitial lung disease with anti-MDA5 antibody-positive dermatomyositis. Clin Rheumatol. (2013) 32:395–8. 10.1007/s10067-012-2142-x 23250474

[29] Kurasawa K Arai S Namiki Y Tanaka A Takamura Y Owada T. Tofacitinib for refractory interstitial lung diseases in anti-melanoma differentiation-associated 5 gene antibody-positive dermatomyositis. Rheumatology (Oxford). (2018) 57:2114–9. 10.1093/rheumatology/key188 30060040

[30] Chen Z Wang X Ye S. Tofacitinib in amyopathic dermatomyositis-associated interstitial lung disease. N Engl J Med. (2019) 381:291–3. 10.1056/NEJMc1900045 31314977

[31] Yu Z Wang L Quan M Zhang T Song H. Successful management with Janus kinase inhibitor tofacitinib in refractory juvenile dermatomyositis: a pilot study and literature review. Rheumatology (Oxford). (2021) 60:1700–7. 10.1093/rheumatology/keaa558 33024992

[32] Takanashi S Kaneko Y Takeuchi T. Tofacitinib in interstitial lung disease complicated with anti-MDA5 antibody-positive dermatomyositis: a literature review. Mod Rheumatol. (2022) 32:231–7. 10.1080/14397595.2021.1906505 33769925

[33] Abe Y Kusaoi M Tada K Yamaji K Tamura N. Successful treatment of anti-MDA5 antibody-positive refractory interstitial lung disease with plasma exchange therapy. Rheumatology (Oxford). (2020) 59:767–71. 10.1093/rheumatology/kez357 31504956

[34] Endo Y Koga T Suzuki T Hara K Ishida M Fujita Y. Successful treatment of plasma exchange for rapidly progressive interstitial lung disease with anti-MDA5 antibody-positive dermatomyositis: a case report. Medicine (Baltimore). (2018) 97:e0436. 10.1097/MD.0000000000010436 29642214 PMC5908626

[35] Shirakashi M Nakashima R Tsuji H Tanizawa K Handa T Hosono Y. Efficacy of plasma exchange in anti-MDA5-positive dermatomyositis with interstitial lung disease under combined immunosuppressive treatment. Rheumatology (Oxford). (2020) 59:3284–92. 10.1093/rheumatology/keaa123 32276271

[36] Fine A Karp JK Peedin AR. The role of therapeutic plasma exchange in clinically amyopathic dermatomyositis with MDA-5 antibody: a case report and review of the literature. J Clin Apher. (2020) 35:483–7. 10.1002/jca.21815 33617011

[37] Gono T Kawaguchi Y Satoh T Kuwana M Katsumata Y Takagi K. Clinical manifestation and prognostic factor in anti-melanoma differentiation-associated gene 5 antibody-associated interstitial lung disease as a complication of dermatomyositis. Rheumatology (Oxford). (2010) 49:1713–9. 10.1093/rheumatology/keq149 20498012

[38] Labrador-Horrillo M Martinez MA. Selva-O'Callaghan A, Trallero-Araguas E, Balada E, Vilardell-Tarres M, et al. Anti-MDA5 antibodies in a large Mediterranean population of adults with dermatomyositis. J Immunol Res. (2014) 2014:290797. 10.1155/2014/290797 24741583 PMC3987881

[39] Li Y Gao X Jia X Zhang X Xu Y Gan Y. Predictors and mortality of rapidly progressive interstitial lung disease in patients with idiopathic inflammatory myopathy: a series of 474 patients. Front Med (Lausanne). (2020) 7:363. 10.3389/fmed.2020.00363 32850886 PMC7412929

[40] Wu W Xu W Sun W Zhang D Zhao J Luo Q. Forced vital capacity predicts the survival of interstitial lung disease in anti-MDA5 positive dermatomyositis: a multi-centre cohort study. Rheumatology (Oxford). (2021) 61:230–9. 10.1093/rheumatology/keab305 33764398

