# Supplementary material for: Mortality Risk Stratification Using Cluster Analysis in Patients With Myositis-Associated Interstitial Lung Disease Receiving Initial Triple-Combination Therapy
Source: Front Med (Lausanne). 2022 May 9;9:883699. doi: 10.3389/fmed.2022.883699 (PMC9124901; doi:10.3389/fmed.2022.883699)
Supplement: Supplementary file 1 [file Data_Sheet_1.docx]

**Supplemental Table S1. Clinical characteristics of patients who initially received triple-combo therapy, stratified by clusters**

| Groups stratified by mortality rates | Low mortality group | | Moderate mortality group | | | High mortality group |
| --- | --- | --- | --- | --- | --- | --- |
| Clusters | Cluster #1  (n = 21) | Cluster #2  (n = 23) | Cluster #3  (n = 87) | Cluster #4  (n = 25) | Cluster #5  (n = 10) | Cluster #6  (n = 19) |
| Demographics |  |  |  |  |  |  |
| Female, no. (%) | 14 (67%) | 16 (70%) | 52 (60%) | 14 (56%) | 5 (50%) | 13 (68%) |
| Age at diagnosis, years | 58 (45-66) | 55 (47-63) | 58 (48-63) | 54 (45-64) | 68 (64-74) | 64 (53-68) |
| Disease duration at diagnosis, months | 2 (1-7) | 1 (1-4) | 2 (1-4) | 2 (1-3) | 2 (1-6) | 1 (1-2) |
| Disease classification |  |  |  |  |  |  |
| PM, no. (%) | 6 (29%) | 0 | 0 | 0 | 3 (30%) | 0 |
| Classic DM, no. (%) | 8 (38%) | 4 (17%) | 0 | 25 (100%) | 6 (60%) | 0 |
| CADM, no. (%) | 7 (33%) | 19 (83%) | 87 (100%) | 0 | 1 (10%) | 19 (100%) |
| Clinical features |  |  |  |  |  |  |
| Fever, no. (%) | 9 (43%) | 13 (57%) | 59 (68%) | 18 (72%) | 6 (60%) | 16 (84%) |
| Raynaud’s phenomenon, no. (%) | 4 (19%) | 1 (4%) | 3 (3%) | 4 (16%) | 0 | 0 |
| Arthritis/arthralgia, no. (%) | 8 (38%) | 13 (57%) | 39 (45%) | 20 (80%) | 5 (50%) | 6 (31%) |
| Skin ulceration, no. (%) | 4 (19%) | 1 (4%) | 19 (22%) | 7 (28%) | 0 | 1 (5%) |
| Laboratory parameters |  |  |  |  |  |  |
| CK, IU/L | 586  (71-1,981) | 102  (73-212) | 135  (75-264) | 334  (121-477) | 1049  (269-3,321) | 140  (43-513) |
| Aldolase, IU/L | 15.4  (7.1-29.9) | 8.5  (6.4-10.9) | 7.9  (5.8-10.3) | 7.8  (6.2-11.8) | 25.7  (10.4-42.4) | 9.1  (6.9-12.7) |
| CRP, mg/dL | 1.1  (0.3-2.4) | 0.6  (0.3-2.1) | 1.0  (0.3-2.0) | 1.2  (0.3-2.3) | 3.6  (1.8-18.4) | 1.9  (1.1-3.6) |
| Ferritin, ng/mL | 183  (94-354) | 223  (132-1,134) | 722  (414-1,325) | 703  (582-1949) | 414  (286-1,389) | 1,530  (612-1,990) |
| KL-6, units/mL | 758  (632-2,262) | 538  (368-1,892) | 746  (562-1,135) | 763  (643-980) | 1,166  (956-2,036) | 1,005  (637-1,493) |
| SP-D, ng/mL | 189  (120-338) | 111  (38-211) | 50  (29-75) | 43  (32-65) | 187  (87-576) | 61  (40-85) |
| Chest HRCT findings |  |  |  |  |  |  |
| Lower consolidation/GGA, no. (%) | 8 (38%) | 18 (78%) | 51 (59%) | 19 (76%) | 8 (80%) | 16 (84%) |
| Lower reticulation, no. (%) | 10 (48%) | 4 (17%) | 19 (22%) | 3 (12%) | 0 | 1 (5%) |
| Random GGA, no. (%) | 1 (5%) | 2 (9%) | 21 (24%) | 4 (16%) | 2 (20%) | 2 (11%) |
| Supplemental oxygen, no. (%) | 0 | 4 (17%) | 0 | 0 | 10 (100%) | 10 (100%) |
| Myositis-specific autoantibodies* |  |  |  |  |  |  |
| Anti-ARS antibody, no. (%) | 21 (100%) | 0 | 0 | 0 | 4 (40%) | 0 |
| Anti-MDA5 antibody, no. (%) | 1 (5%) | 0 | 87 (100%) | 25 (100%) | 2 (20%) | 19 (100%) |

Continuous variables are shown as the median (25-75 percentile).

**Two patients had both anti-ARS and anti-MDA5 antibodies.

PM, polymyositis; CADM, clinically amyopathic dermatomyositis; CK, creatine kinase; CRP, C-reactive protein; KL-6, Krebs von den Lungen-6; SP-D, surfactant protein-D; HRCT, high-resolution computed tomography; GGA, ground-glass attenuation; anti-ARS, anti-aminoacyl tRNA synthetase; anti-MDA5, anti-melanoma differentiation-associated gene 5.

**Supplemental Figure S1.** Predictor importance of individual variables used for applying the cluster model to patients who initially received dual-combo therapy or monotherapy. A predictor importance ≥ 0.4 indicates a significant variable. Anti-MDA5, anti-melanoma differentiation-associated gene 5; Anti-ARS, anti-aminoacyl transfer RNA synthetase; CADM, clinically amyopathic dermatomyositis; CRP, C-reactive protein; KL-6, Krebs von den Lungen-6.

**Supplemental Table S2. Clinical characteristics of anti-MDA5 antibody-positive patients who initially received triple-combo therapy, stratified by clusters**

| Clusters | Cluster #M1  (n = 87) | Cluster #M2  (n = 26) | Cluster #M3  (n = 21) |
| --- | --- | --- | --- |
| Demographics |  |  |  |
| Female, no. (%) | 52 (60%) | 15 (58%) | 14 (68%) |
| Age at diagnosis, years | 58 (48-63) | 54 (44-64) | 65 (54-69) |
| Disease duration at diagnosis, months | 2 (1-4) | 2 (1-3) | 1 (1-1) |
| Disease classification |  |  |  |
| PM, no. (%) | 0 | 0 | 0 |
| Classic DM, no. (%) | 0 | 26 (100%) | 2 (10%) |
| CADM, no. (%) | 87 (100%) | 0 | 19 (90%) |
| Clinical features |  |  |  |
| Fever, no. (%) | 59 (68%) | 18 (69%) | 18 (86%) |
| Raynaud’s phenomenon, no. (%) | 3 (3%) | 4 (17%) | 0 |
| Arthritis/arthralgia, no. (%) | 39 (45%) | 20 (77%) | 7 (39%) |
| Skin ulceration, no. (%) | 19 (22%) | 7 (27%) | 1 (5%) |
| Laboratory parameters |  |  |  |
| CK, IU/L | 135 (75-264) | 355 (130-537) | 157 (50-576) |
| Aldolase, IU/L | 7.9 (5.8-10.3) | 7.9 (6.3-11.9) | 9.1 (7.1-13.2) |
| CRP, mg/dL | 1.0 (0.3-2.0) | 1.0 (0.3-2.2) | 1.9 (1.1-3.7) |
| Ferritin, ng/mL | 722 (414-1,325) | 679 (421-1.832) | 1,270 (644-2,064) |
| KL-6, units/mL | 746 (562-1,135) | 763 (658-979) | 1,010 (684-1,357) |
| SP-D, ng/mL | 50 (29-75) | 43 (33-73) | 61 (41-90) |
| Chest HRCT findings |  |  |  |
| Lower consolidation/GGA, no. (%) | 51 (59%) | 19 (73%) | 18 (86%) |
| Lower reticulation, no. (%) | 19 (22%) | 4 (15%) | 1 (5%) |
| Random GGA, no. (%) | 21 (24%) | 4 (15%) | 2 (10%) |
| Supplemental oxygen, no. (%) | 0 | 0 | 10 (100%) |
| Mortality, no. (%) | 30 (35%) | 9 (35%) | 20 (95%) |

Continuous variables are shown as the median (25-75 percentile).

PM, polymyositis; CADM, clinically amyopathic dermatomyositis; CK, creatine kinase; CRP, C-reactive protein; KL-6, Krebs von den Lungen-6; SP-D, surfactant protein-D; HRCT, high-resolution computed tomography; GGA, ground-glass attenuation; anti-ARS, anti-aminoacyl tRNA synthetase; anti-MDA5, anti-melanoma differentiation-associated gene 5.

**Supplemental Table S3. Comparison of sex and explanatory variables used for the cluster analysis between triple-combo therapy and dual-combo therapy/monotherapy in individual clusters***

| Dual combo/monotherapy | Cluster #1 | Cluster #2 | Cluster #3 | Cluster #4 | Cluster #5 | Cluster #6 |
| --- | --- | --- | --- | --- | --- | --- |
| Number of patients in  triple combo, dual combo/mono, no. | 21/126 | 23/86 | 87/43 | 25/15 | 10/10 | 19/3 |
| Female, no. (%) |  |  |  |  |  |  |
| Triple combo | 14 (67%) | 16 (70%) | 52 (60%) | 14 (56%) | 5 (50%) | 13 (68%) |
| Dual combo/mono | 93 (74%) | 54 (63%) | 31 (70%) | 10 (67%) | 5 (56%) | 1 (33%) |
| *P* values | 0.50 | 0.54 | 0.23 | 0.51 | 0.81 | 0.53 |
| Age at diagnosis, years |  |  |  |  |  |  |
| Triple combo | 58 (45-66) | 55 (47-63) | 58 (48-63) | 54 (45-64) | 68 (64-74) | 64 (53-68) |
| Dual combo/mono | 53 (45-64) | 61 (52-67) | 48 (39-59) | 47 (37-60) | 69 (62-77) | 72 (59-ND) |
| *P* values | 0.61 | 0.19 | 0.001 | 0.17 | 1.0 | 0.27 |
| CADM, no. (%) |  |  |  |  |  |  |
| Triple combo | 7 (33%) | 19 (83%) | 87 (100%) | 0 | 1 (10%) | 19 (100%) |
| Dual combo/mono | 46 (37%) | 22 (26%) | 43 (100%) | 0 | 6 (60%) | 3 (100%) |
| *P* values | 0.78 | <0.001 | 1.0 | 1.0 | 0.06 | 1.0 |
| CRP, mg/dL |  |  |  |  |  |  |
| Triple combo | 1.1  (0.3-2.4) | 0.6  (0.3-2.1) | 1.0  (0.3-2.0) | 1.2  (0.3-2.3) | 3.6  (1.8-18.4) | 1.9  (1.1-3.6) |
| Dual combo/mono | 0.8  (0.2-2.1) | 0.3  (0.1-1.2) | 0.3  (0.1-1.2) | 0.2  (0.1-1.2) | 3.4  (1.6-18.5) | 3.1  (0.4-ND) |
| *P* values | 0.46 | 0.22 | 0.005 | 0.09 | 1.0 | 0.53 |
| KL-6, units/ml |  |  |  |  |  |  |
| Triple combo | 758  (632-2,262) | 538  (368-1,892) | 746  (562-1,135) | 763  (643-980) | 1,166  (956-2,036) | 1,005  (637-1,493) |
| Dual combo/mono | 927  (563-1,666) | 662  (386-1,131) | 707  (547-1,148) | 922  (630-1,370) | 1,353  (711-2,406) | 472  (338-ND) |
| *P* values | 0.25 | 0.51 | 0.58 | 0.15 | 1.0 | 0.16 |
| Supplemental oxygen, no. (%) |  |  |  |  |  |  |
| Triple combo | 0 | 4 (17%) | 0 | 0 | 10 (100%) | 10 (100%) |
| Dual combo/mono | 0 | 0 | 0 | 0 | 9 (100%) | 3 (100%) |
| *P* values | 1.0 | 0.002 | 1.0 | 1.0 | 1.0 | 1.0 |
| Anti-ARS antibody*, no. (%) |  |  |  |  |  |  |
| Triple combo | 21 (100%) | 0 | 0 | 0 | 4 (40%) | 0 |
| Dual combo/mono | 126 (100%) | 0 | 0 | 0 | 6 (67%) | 0 |
| *P* values | 1.0 | 1.0 | 1.0 | 1.0 | 1.0 | 1.0 |
| Anti-MDA5 antibody*, no. (%) |  |  |  |  |  |  |
| Triple combo | 1 (5%) | 0 | 87 (100%) | 25 (100%) | 2 (20%) | 19 (100%) |
| Dual combo/mono | 0 | 0 (0) | 44 (100%) | 15 (100%) | 0 | 3 (100%) |
| *P* values | 1.0 | 1.0 | 1.0 | 1.0 | 0.47 | 1.0 |

Continuous variables are shown as the median (25-75 percentile). Comparisons between two groups were made using the chi-square test, Fisher’s exact test or the Mann–Whitney U test if applicable.

*Two patients had both anti-ARS and anti-MDA5 antibodies.

CADM, clinically amyopathic dermatomyositis; anti-ARS, anti-aminoacyl transfer RNA synthetase; anti-MDA5, anti-melanoma differentiation-associated gene 5; CRP, C-reactive protein; KL-6, Krebs von den Lungen-6; ND, not determinate.
